# Supplementary material for: Optically oriented attachment of nanoscale metal-semiconductor heterostructures in organic solvents via photonic nanosoldering
Source: Nat Commun. 2019 Oct 30;10:4942. doi: 10.1038/s41467-019-12827-w (PMC6821866; doi:10.1038/s41467-019-12827-w)
Supplement: Supplementary file 2 — Supplementary Information [file 41467_2019_12827_MOESM2_ESM.docx]

Supplementary Information: Optically Oriented Attachment of Nanoscale Metal-Semiconductor Heterostructures in Organic Solvents via Photonic Nanosoldering

M. Crane, E. Pandres et al.

**Supplementary Note 1: Bismuth-Seeded Germanium Nanowire Synthesis**

Briefly, bismuth nanocrystals were synthesized by first generating tris[bis(trimethylsilyl)amido] bismuth^1^ and then heating to 200°C in the presence of sodium bis(trimethylsilyl)amide in a stabilizing solution of 1-octadecene containing 25 wt.% poly(1-hexadecene-co-1-vinylpyrrolidone).^2^ The nanocrystal dispersion was transferred to a nitrogen atmosphere and washed three times by centrifuging with a 2:3 ratio of toluene:ethanol at 5230 RCF to remove excess polymer, which is known to facilitate the development of branches during germanium nanowire growth.^3^ The cleaned bismuth nanocrystals were dispersed and stored in anhydrous toluene. Germanium nanowires were then synthesized via thermal decomposition of diphenylgermane (DPG) at 350°C in the presence of bismuth nanocrystals dispersed in a mixture of high boiling point solvents (1:1.7 trioctylphosphine oxide and squalane) to facilitate anisotropic, solution-liquid-solid (SLS) germanium nanowire growth.^3^

**Supplementary Note 2: Off-Axis Nanosoldering**

During attempts to trap and assemble nanowires dispersed in toluene, nanowires accelerated toward the trap at higher velocities than in squalane and occasionally fused at an angle, rather than coaxially. This resulted in the binding of nanowires at angles, yielding radially-asymmetric structures that eventually rotated out of the trap. While impractical for the continued production of linear periodic heterostructures, this does illustrate the potential application of the nanosoldering process to create more complicated structures.^4^ One explanation for this phenomenon is the massive disparity between the viscosity of squalane (28.4 cP) and toluene (0.58 cP). The hydrodynamic drag force in toluene is three orders of magnitude lower than the drag force in squalane; consequently, the radiation pressure required to align and push the nanowire into the optical trap is three orders of magnitude higher in squalane than in toluene. For the Gaussian beam profile used in the single-beam optical trap, there exists a much smaller solid angle over which this balance of forces is satisfied in squalane than in toluene. Thus, nanowires in predominately squalane solutions are only aligned and trapped if they are almost directly below the optical trap—that is, if $\theta\approx0$. However, in toluene, radiation pressure and torque will act over a much wider solid angle ($\theta>0)$ to drive nanowires into the optical trap, which can enable off-axis addition. Another potential challenge is that, given the low Reynolds number of the system, nanowires enter the trap at their terminal velocity, which is determined by the viscosity of the solvent and the laser power.^5^ The large difference in viscosity between squalane and toluene illustrates that nanowires will approach the trap at much higher velocities in toluene than in squalane, which may prevent optical alignment prior to nanosoldering. Thus, using a viscous solvent enables oriented alignment by decreasing the solid angle of radiation pressure, thereby restricting the volume and angle of nanowires approaching from below the optical trap. In addition, using a high power may be required to impart large radiation pressure forces to align the nanowire prior to entering the trap. Alternatively, heating-induced hydrodynamic effects could also facilitate nanowire alignment.

**Supplementary Note 3: Laser-Induced Heating During Optical Trapping in Organic Media**

When employing water-based optical trapping with a near-infrared (NIR) laser, the relatively large absorption coefficient of water can lead to appreciable adventitious solvent heating. For temperature-sensitive measurements, this effect has led to the use of D_2_O as a trapping medium due to its low NIR absorption coefficient.^6^ A number of groups have independently studied photothermal solvent effects and have measured heating on the order of ~1–10 K/W.^7,8^ As a potential alternative, organic solvents often exhibit significantly lower NIR absorption coefficients and thermal conductivities.^9^ For an empty optical trap, the dominant heat dissipation mechanism is thermal conduction. Using reported NIR absorption coefficients, α, and thermal conductivities, λ, we estimate that the solvent-induced heating in toluene (α = 3.5 m^-1^, λ = 0.14 W·m^-1^K^-1^) is 1.7 W/mK.^7^ A similar analysis for water (α = 29 m^-1^, λ = 0.60 W·m^-1^K^-1^) yields 3.6 W/mK, suggesting that, for an empty trap, solvent-induced heating effects are slightly lower in organic solvents than in aqueous solvents. However, when the optical trap contains a highly absorbing particle, the lower thermal conductivity of organic solvents (~0.15 W·m^-1^K^-1^) leads to significantly greater local temperatures. The powers reported in the manuscript are measured after beam expansion and before the high numerical aperture trapping objective, which decreases the laser power by less than 30% at these trapping wavelengths.

**Supplementary Note 4: Back Focal Plane Interferometry and Temperature Calculations**

The optical trapping kit was purchased from Thor Labs and modified with a different condenser. During trapping, forward-scattered light was collected with a long-working-distance 10X Mitutoyo condenser (Plan Apo infinity-corrected long WD objective, stock no. 46–144) and focused onto a quadrant photodiode, providing a measurement of the trapped nanowire position. The quadrant photodiode data was collected with a DAQ card (PCIe-6361 X series, National Instruments) and controlled with home-built LabVIEW software.^10^ Power spectra were collected and fit to solve for the diffusion coefficient. These diffusion coefficients were then used to calculate the local temperature at either the hydrodynamic radius of the particle or at the surface of the particle using a hot Brownian motion correlation. The instrument and methodology described here are outlined in previous reports in more detail.^6,10,11^

**Supplementary Note 5: Heat Transport Calculations**

All of the below calculations included iterative temperature-dependent heat transfer boundary conditions; we also allowed the bismuth nanocrystal tip to vary in composition as a function temperature. The source term was calculated with a discrete dipole approximation by applying DDSCAT code in Matlab to a nanostructure being illuminated in the +Z direction, shown in Figure 4a of the main text.

To solve the heat transport equation, we nondimensionalized Equation 1 to

| $\frac{\partial\theta}{\partial\tau}=\frac{1}{\xi}\frac{\partial}{\partial\xi}\left( \xi\frac{\partial\theta}{\partial\xi} \right)+a^{2}\frac{\partial^{2}\theta}{\partial\zeta^{2}}+\sigma$ | (1) |
| --- | --- |

where the variables are defined as

| $\theta=\frac{T-T_{\infty}}{T_{\infty}}, \tau= \frac{\alpha t}{R^{2}}, \xi= \frac{r}{R}, \zeta= \frac{z}{L}, \sigma= \frac{SR^{2}}{\kappa T_{\infty}}, a= \frac{R}{L}.$ | (2a-f) |
| --- | --- |

Here, $T_{\infty}$, $S$, $R$, $L$, and $\kappa$ are the bulk fluid temperature, source function from DDSCAT, radius and length of the overall nanowire heterostructure, and thermal conductivity of either bismuth or germanium, respectively. The thermal diffusivity, $\alpha$, is defined as $\kappa/{(\rho C_{p})}$, where $\rho$ and $C_{p}$ are the density and heat capacity of the nanowire. The values used for these parameters and all others used in the calculation are presented in Supplementary Table 1. The DDSCAT simulations demonstrated almost no dependence of the source term on $\phi$, and we neglect the $\phi$-dependence of the Laplacian. In addition, there is very little variation of the source term radially and axially within the bismuth and germanium portions of the heterostructure. Thus, we employed volume-average sources for the bismuth nanocrystal ($\sigma_{Bi}$) and germanium nanowire ($\sigma_{Ge})$. Details of the DDSCAT calculation and temperature dependence are discussed below.

In addition to the modifications discussed in the main text, we employed a temperature-dependent boundary condition via the heat transfer coefficient, $h$, to account for convective heat loss at the nanowire surface. We defined the heat transfer coefficient as follows

| $h=\frac{k}{L}\left( \frac{4}{3}\left( \frac{7RaPr}{5\left( 20+21Pr \right)} \right)^{\frac{1}{4}}+\frac{4}{35}\left( \frac{\left( 272+315Pr \right)L}{\left( 64+63Pr \right)2r} \right) \right)$ | (3) |
| --- | --- |

where $L$ is nanowire length, $r$ is the nanowire radius, $h$ is the heat transfer coefficient, and $k$ is the thermal conductivity of the medium.^12,13^ Here, $Ra$, the Rayleigh number, is the product of the Grashof, $Gr$, and Prandtl, $Pr$, numbers,

| $Gr= \frac{g\beta\Delta TL^{3}}{\mu^{2}} \text{and} Pr = \frac{\mu\rho C_{p}}{k}$ | (4) |
| --- | --- |

where $g$, $\beta$, $\rho$, and $C_{p}$ are the acceleration due to gravity, the thermal expansion coefficient, density, and heat capacity of the medium evaluated at the film temperature, $\left( T_{surface, avg}+T_{\infty} \right)/2$. Here, $\Delta T$ is the temperature difference between the nanowire surface and the bulk solvent. The temperature-dependent properties of squalane are shown in Supplementary Figure 1.^14^


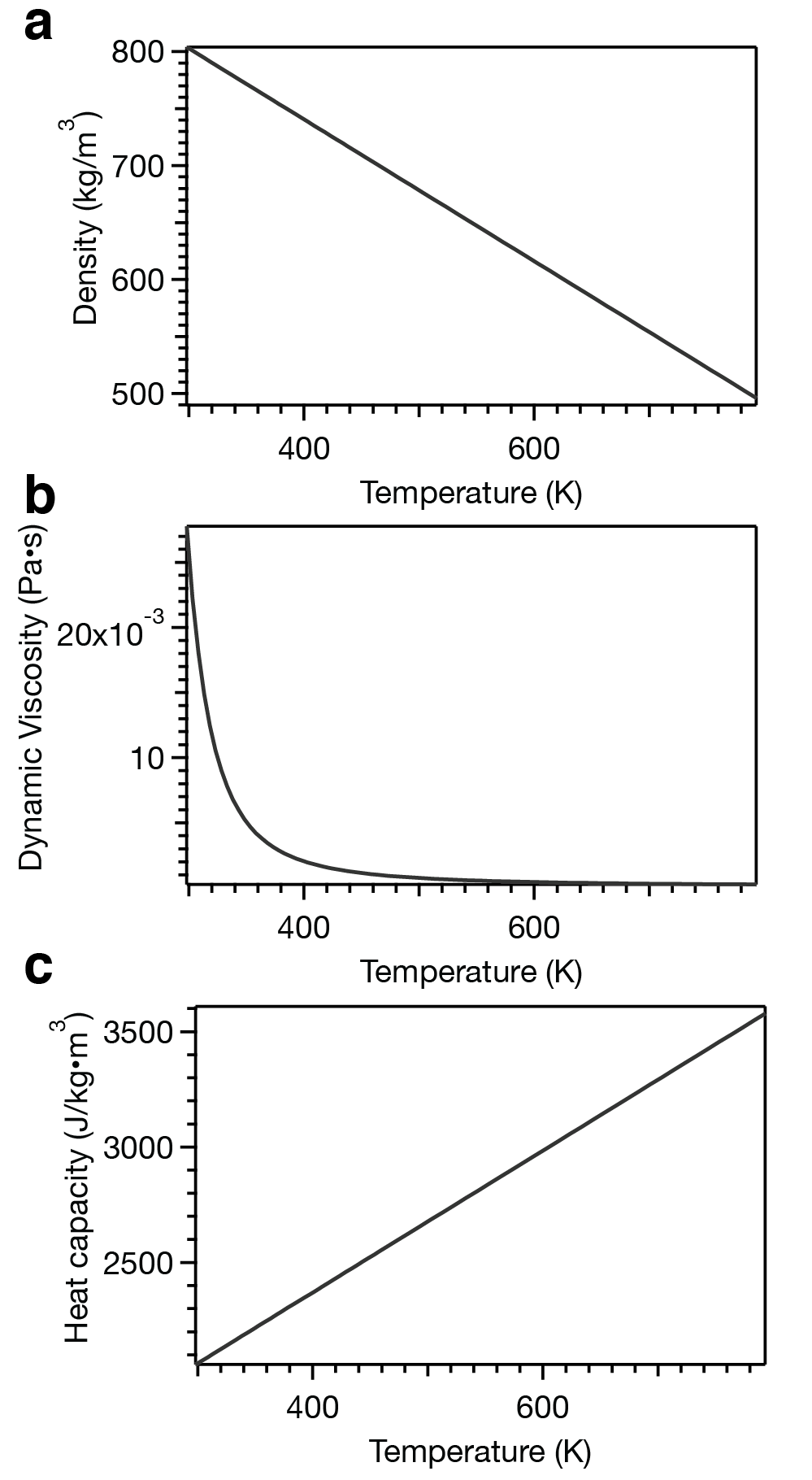


**Supplementary Figure 1.** Temperature-dependent properties of squalane used in the heat transfer calculation including the density (a), dynamic viscosity (b), and heat capacity (c). While none of the calculations exceeded this temperature range, the code bounded the values so that any iteration that produced temperatures above 793 K would default to the solvent properties at 793 K instead.

The radial boundary conditions were then implemented as

| $\left. \frac{\partial\theta}{\partial\xi} \right\vert_{\xi=0}=bounded \mathrm{and} \left. \frac{\partial\theta}{\partial\xi} \right\vert_{\xi=1}=-{Bi}_{R}\left. \theta\right\vert_{\xi=1}= -\frac{hR}{\kappa}\left. \theta\right\vert_{\xi=1}.$ | (S5) |
| --- | --- |

The axial boundary conditions were then defined via Fourier’s law for conduction to the bottom and top glass coverslips at $T_{\infty}$ as

| $\left. \frac{\partial\theta}{\partial\xi} \right\vert_{\zeta=1}=-\frac{L_{chamber}\kappa_{f}}{\kappa_{B}}\left. \theta\right\vert_{\zeta=1} \mathrm{and} \left. \frac{\partial\theta}{\partial\xi} \right\vert_{\zeta=0}=\frac{L_{chamber}\kappa_{f}}{\kappa_{G}}\left. \theta\right\vert_{\zeta=0}$ | (6) |
| --- | --- |

where $L_{chamber}$ is half the height of the chamber (50 µm) and $\kappa_{f}$, $\kappa_{B}$, and $\kappa_{G}$ are the thermal conductivities of squalane (0.15 W·m^-1^K^-1^), the bismuth nanocrystal (3 W·m^-1^K^-1^),^15^ and the germanium nanowire (7 W·m^-1^K^-1^), respectively.^16^

Using a classical product solution, we first solve the homogenous equation

| $\frac{\partial\theta}{\partial\tau}=\frac{1}{\xi}\frac{\partial}{\partial\xi}\left( \xi\frac{\partial\theta}{\partial\xi} \right)+a^{2}\frac{\partial^{2}\theta}{\partial\zeta^{2}}$ | (7) |
| --- | --- |

by assuming a solution of the form

| $\theta\left( \tau,\xi,\zeta\right)=A_{m,n}\left( \tau\right)X_{m}\left( \xi\right)Z_{n}\left( \zeta\right).$ | (8) |
| --- | --- |

The radial equation produces

| $X_{m}\left( \xi\right)= J_{0}\left( \mu_{m}\xi\right)$ | (9) |
| --- | --- |

with eigenvalues satisfied by

| $\mu_{m}J_{1}\left( \mu_{m} \right)={Bi}_{R}J_{0}\left( \mu_{m} \right)$ | (10) |
| --- | --- |

where $J_{\alpha}$ is a Bessel function of order $\alpha$. Similarly, the axial solution yields

| $Z_{n}\left( \zeta\right)=cos\left( \gamma_{n}\zeta\right)+\frac{{Bi}_{0}}{\gamma_{n}}sin\left( \gamma_{n}\zeta\right)$ | (11) |
| --- | --- |

with eigenvalue equation

| $\left( \gamma_{n}^{2}-{Bi}_{0}{Bi}_{1} \right)sin\left( \gamma_{n} \right)= \left( {Bi}_{0}+ {\gamma_{n}Bi}_{1} \right)cos\left( \gamma_{n} \right).$ | (12) |
| --- | --- |

The Biot number, $Bi$, is defined as the ratio of heat transport away from the nanostructure to the heat transport within an individual nanostructure. Here, the Biot numbers, are defined as

| ${Bi}_{0}=\frac{hL}{\kappa_{G}}= \frac{L\kappa}{L_{chamber}\kappa_{G}}, {Bi}_{1}=\frac{hL}{\kappa_{B}}= \frac{L\kappa}{L_{chamber}\kappa_{B}}, {Bi}_{R}=\frac{hR}{\kappa_{R}} ,$ | (13) |
| --- | --- |

where $L_{chamber}$ is the distance from the nanowire to the coverslip, 50 µm.

We combine these to define a product solution

| $\theta\left( \tau,\xi,\zeta\right)=\sum_{m} \sum_{n} A_{m,n}\left( \tau\right)X_{m}\left( \xi\right)Z_{n}\left( \zeta\right).$ | (14) |
| --- | --- |

To solve for the coefficients $A_{m,n}\left( \tau\right)$, we substitute this solution into the full inhomogeneous equation (Eq. 1) as

| $\sum_{m} \sum_{n} \left( \frac{\partial A_{m,n}\left( \tau\right)}{\partial\tau}+\gamma_{n}^{2}A_{m,n}\left( \tau\right) \right)X_{m}\left( \xi\right)Z_{n}\left( \zeta\right)= \sigma.$ | (15) |
| --- | --- |

Here, we’ve defined $\lambda_{m,n}^{2}= \mu_{m}^{2}+a^{2}\gamma_{n}^{2}$. Because Eq. 15 is a Sturm-Liouville equation, the solutions $X_{m}\left( \xi\right)$ and $Z_{n}\left( \zeta\right)$ are orthogonal. Thus, we can apply orthogonality as

| $\left( \frac{\partial A_{m,n}\left( \tau\right)}{\partial\tau}+\gamma_{n}^{2}A_{m,n}\left( \tau\right) \right)\left\vert\left\vert X_{m} \right\vert\right\vert^{2}\left\vert\left\vert Z_{n} \right\vert\right\vert^{2}=\int_{0}^{1} \int_{0}^{1} \sigma\xi^{'}X_{m}\left( \xi^{'} \right)Z_{n}\left( \zeta^{'} \right)d\xi^{'}d\zeta^{'}.$ | (16) |
| --- | --- |

where $\xi^{'}$ and $\zeta^{'}$ are dummy variables for integration and $\left| \left| X_{m} \right| \right|^{2}$ and $\left| \left| Z_{n} \right| \right|^{2}$ are normalization factors defined by

| $\left\vert\left\vert X_{m} \right\vert\right\vert^{2}=\int_{0}^{1} \xi^{'}\left[ J_{0}\left( {\mu_{m}\xi}^{'} \right) \right]^{2}{d\xi}^{'}$ | (17) |
| --- | --- |

and

| $\left\vert\left\vert Z_{n} \right\vert\right\vert^{2}=\int_{0}^{1} \left[ Z_{n}\left( \zeta^{'} \right) \right]^{2}{d\zeta}^{'}.$ | (18) |
| --- | --- |

To solve for the coefficients $A_{m,n}\left( \tau\right)$, we then apply an integrating factor and take the limit as $\tau$ approaches infinity, yielding the solution

| $A_{m,n}= \frac{\left( 1-e^{-\lambda_{m,n}^{2}} \right)}{\lambda_{m,n}^{2}}\int_{0}^{1} \int_{0}^{1} {\sigma\xi}^{'}\frac{X_{m}\left( \xi^{'} \right)}{\left\vert\left\vert X_{m} \right\vert\right\vert^{2}}\frac{Z_{n}\left( \zeta^{'} \right)}{\left\vert\left\vert Z_{n} \right\vert\right\vert^{2}}d\xi^{'}d\zeta^{'}$ | (19) |
| --- | --- |

Due to its weak radial dependence, the source, $\sigma$, only varies axially. In addition, within the bismuth and germanium, it has very little axial dependence, and we can simplify the equation as

| $A_{m,n}= \frac{\left( 1-e^{-\lambda_{m,n}^{2}} \right)}{\lambda_{m,n}^{2}}\int_{0}^{1} \xi^{'}\frac{X_{m}\left( \xi^{'} \right)}{\left\vert\left\vert X_{m} \right\vert\right\vert^{2}}d\xi^{'}\left[ \sigma_{Ge}\int_{0}^{\beta} \frac{Z_{n}\left( \zeta^{'} \right)}{\left\vert\left\vert Z_{n} \right\vert\right\vert^{2}}d\zeta^{'}+ \sigma_{Bi}\int_{\beta}^{1} \frac{Z_{n}\left( \zeta^{'} \right)}{\left\vert\left\vert Z_{n} \right\vert\right\vert^{2}}d\zeta^{'} \right]$ | (20) |
| --- | --- |

where $\beta$ is the nondimensional axial position of the bismuth-germanium interface. The temperature of the heterostructure can then be solved by Eq. 14.

**DDSCAT Solution:**

To calculate the source function, we used the discrete dipole approximation (DDA) implemented in DDSCAT. We created the cylinder-cylinder heterostructure in MATLAB, using ~87,000 dipoles. The discrete dipole approximation is valid for^17^

| $\left( \frac{3V}{4\pi} \right)^{\frac{1}{3}}< \frac{\lambda}{\left\vert m \right\vert}\left( \frac{N}{{10}^{6}} \right)^{\frac{1}{3}}$ | (21) |
| --- | --- |

where $V$, $N$, and $\lambda$ are the volume of the structure in µm^3^, the number of dipoles, and the wavelength in µm, and $m$ is the ratio of the refractive index of the nanomaterial to that of the solvent. For a cylinder-cylinder heterostructure with a diameter of 32 nm, an overall length of 1000 nm, and a bismuth cylinder tip with a length of 40 nm ($\beta=0.96$). For the computationally complex case, assuming that the entire cylinder is bismuth with n_Bi_ = 4.33 – 3.88i and n_squalane_ = 1.48, the use of 87,000 dipoles (19 dipoles across the diameter and 297 dipoles along the length of the wire) satisfies this inequality.

**Temperature-Dependent Nanocrystal Characteristics:**

Due to the high temperature of the bismuth seed and its low melting point, the bismuth nanocrystal at the end of the nanowire heats, melts, and alloys with the germanium during nanosoldering. To account for this effect, we allowed the composition of the nanocrystal to vary as a function of temperature according to the Bi-Ge binary phase diagram, as shown in Supplementary Figure 2.

**Supplementary Figure 2.** Temperature-dependent bismuth concentration in the bismuth-germanium binary alloy system.^18^

This allows the composition of the molten tip to vary from 100% bismuth below 271°C to 15% bismuth at 920°C. To approximate the effect of alloying on the bismuth nanocrystal, we employed an effective medium approximation^19^ using the atomic volumes of bismuth and germanium. This allows us to define a temperature-dependent complex refractive index (Supplementary Figure 3).

**Supplementary Figure 3.** Temperature dependence of the imaginary and real components of the bismuth alloy refractive index. To calculate these values, we used the volume-averaged refractive index of bismuth and germanium, weighted by the temperature-dependent concentrations in Supplementary Figure 2.

As the temperature of the bismuth tip increases, the concentration of germanium in the bismuth alloy increases, causing Im{n_Bi_} to decrease and Re{n_Bi_} to increase. Using DDSCAT, we calculated the internal electric field profile for the cylinder-cylinder heterostructure. To simulate the effects of melting and alloying, we used the refractive index values from Supplementary Figure 3. These normalized, volume-averaged results are presented in Supplementary Figure 4.

**Supplementary Figure 4.** The volume-averaged, normalized electric field in the germanium and bismuth portions of the nanowire heterostructure. These were calculated by using DDSCAT to evaluate the internal electric fields in the heterostructure. At each temperature, the bismuth alloy refractive index was set using Supplementary Figure 3, allowing it to vary as the composition of the metal alloy tip changes with temperature. Because the composition of the germanium section of the nanowire does not change significantly with temperature, we do not incorporate any temperature dependence.

We then convert these data into a source function, $\dot{Q}^{'''}$, for the heat transfer equation (Equation 2), as shown in Supplementary Figure 5.

**Supplementary Figure 5.** The electric-field-normalized, temperature-dependent source function calculated from the volume-averaged internal fields in Supplementary Figure 4 and Equation 2 in the main text.

As expected from Equation 2, the bismuth metal alloy source term decreases as its temperature rises and it incorporates additional germanium with a lower absorption coefficient. For example, the product of Re{n_Bi_} and Im{n_Bi_} decreases from 16.8 at 271°C to 3.26 at 920°C.

To incorporate these temperature-dependent effects, we calculated the average temperature of the heterostructure and then iterate until the converged to within 1% of the previous average temperature by allowing only temperature-dependent solvent properties to vary. Then, we calculated the average temperature of the bismuth nanocrystal tip, updated the new source function as described above, and then iterated with fixed solvent properties until the average temperature of the nanocrystal converged to within 1% of the average temperature of the previous iteration.

**Supplementary Figure 6.** The calculated trap stiffness as a function of power before beam expansion.^6,20^ Note that this includes the temperature-dependent solvent properties shown in Figure 4. The significant variation in viscosity with trapping power strongly influences the trap stiffness. Error bars indicate the standard deviation from three separate measurements.

**
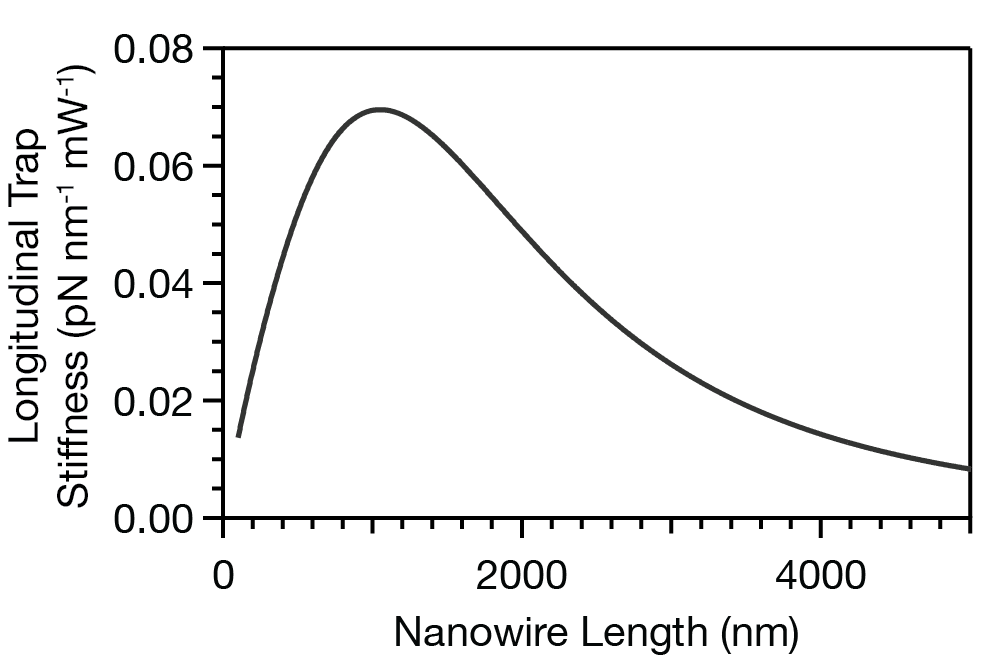
**

**Supplementary Figure 7.** Analytical approximation of nanowire longitudinal trap stiffness as a function of length, using the theory developed by Simpson and Hanna.^21^ To include the effect of nanowire length, we used the Maxwell-Garnett approximation weighed by the volume fraction of the bismuth nanocrystal, which remains constant, and the germanium nanowire, which varies with length. In the first region, the longitudinal trap stiffness increased as the length of that nanowire increased up to ~1200 nm—that is, as the distance from focal plane to the bismuth nanocrystal increased. This reflects the observation that longer nanowires are easier to trap than shorter nanowires. In the second region, the trap stiffness decreased due to a decreased magnitude of the gradient force as more of the nanowire sits outside of the focal plane.


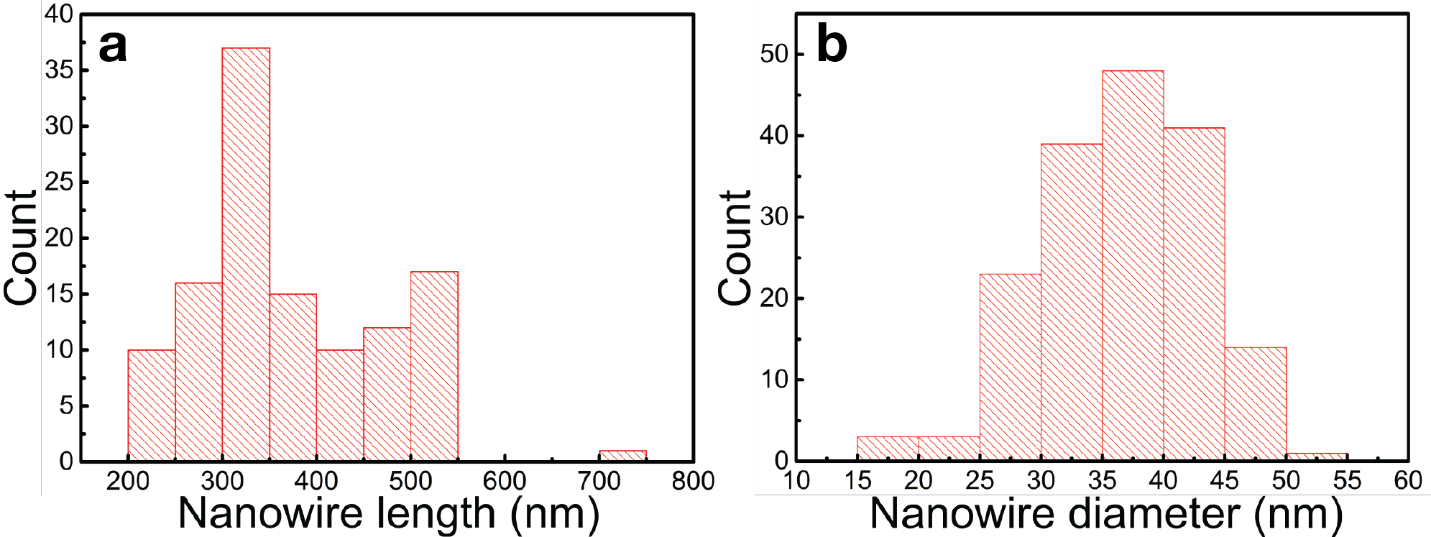

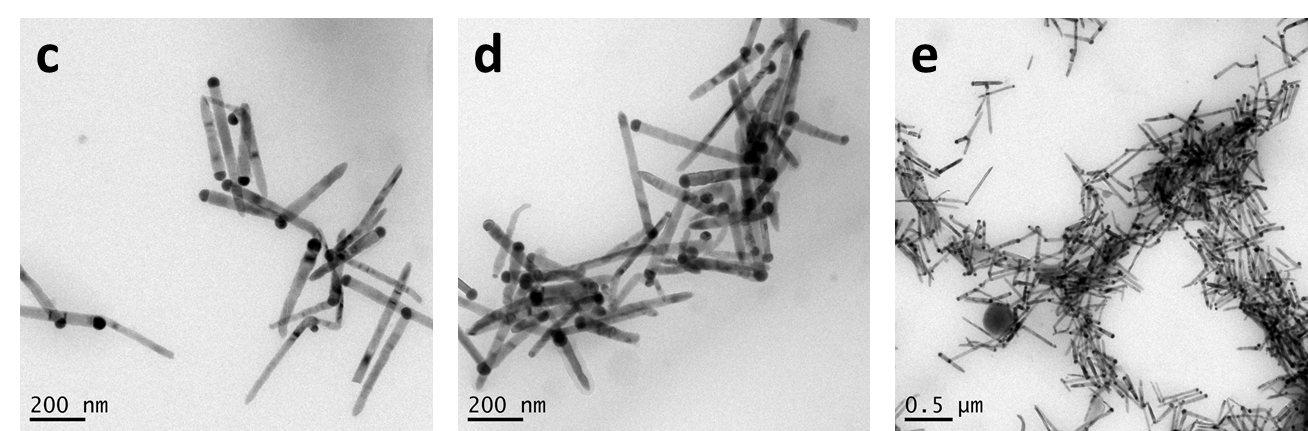


**Supplementary Figure 8.** Length and diameter distributions (a,b) for the bismuth-seeded germanium nanowires shown in Figure 1 of the main text. Additional representative TEM images (c-e), illustrating the monodispersity of the nanowire building blocks. The nanowires were synthesized by a solution-liquid-solid growth that can be easily tuned to create a wide range of lengths and diameters.

**Supplementary Table 1.** Parameters used for heat transport calculations^14,22–24^.

| Variable | Value | Units |
| --- | --- | --- |
| $T_{\infty}$ | 298 | K |
| $R$ | 16 | nm |
| $L$ | 1000 | nm |
| $\beta$ | 0.96 | - |
| $\kappa_{Bi}$ | 3 | W·m^-1^K^-1^ |
| $\kappa_{Ge}$ | 7.5 | W·m^-1^K^-1^ |
| $\alpha_{Bi}$ | 2.51•10^-5^ | m^2^/s |
| $\alpha_{Ge}$ | 4.38•10^-6^ | m^2^/s |
| $N_{Squalane}$ | 1.4818 |  |
| $N_{Ge}$ | 4.42 - 0.12i | - |
| $N_{Bi}$* | 3.88 - 4.33i | - |
| $\mu_{perm}$ | 1.2566•10^-6^ | N/A^2^ |
| $c$ | 299790000 | m/s |
| $\varepsilon$ | 8.8542•10^-12^ | F/m |
| $\epsilon$ | 1 | - |
| $\lambda$ | 1020 | nm |
| ${Bi}_{0}$ | 3.73•10^-4^ | - |
| ${Bi}_{1}$ | 9.33•10^-4^ | - |
| ${Bi}_{R}*$ | 6.36•10^-3^ | - |
|  |  |  |

* Initial values used for temperature-dependent parameters.

**Supplementary Information References**

1. Carmalt, C. J. *et al.* Homoleptic Bismuth Amides. in *Inorganic Syntheses* 98–101 (Wiley-Blackwell, 2007). doi:10.1002/9780470132623.ch15

2. Wang, F., Tang, R., Yu, H., Gibbons, P. C. & Buhro, W. E. Size- and Shape-Controlled Synthesis of Bismuth Nanoparticles. *Chem. Mater.* **20**, 3656–3662 (2008).

3. Chockla, A. M., Harris, J. T. & Korgel, B. A. Colloidal Synthesis of Germanium Nanorods. *Chem. Mater.* **23**, 1964–1970 (2011).

4. Galajda, P. & Ormos, P. Rotors produced and driven in laser tweezers with reversed direction of rotation. *Appl. Phys. Lett.* **80**, 4653–4655 (2002).

5. Ashkin, A. Acceleration and Trapping of Particles by Radiation Pressure. *Phys. Rev. Lett.* **24**, 156–159 (1970).

6. Roder, P. B., Smith, B. E., Zhou, X., Crane, M. J. & Pauzauskie, P. J. Laser refrigeration of hydrothermal nanocrystals in physiological media. *PNAS* **112**, 15024–15029 (2015).

7. Peterman, E. J. G., Gittes, F. & Schmidt, C. F. Laser-Induced Heating in Optical Traps. *Biophysical Journal* **84**, 1308–1316 (2003).

8. Kyrsting, A., Bendix, P. M., Stamou, D. G. & Oddershede, L. B. Heat Profiling of Three-Dimensionally Optically Trapped Gold Nanoparticles using Vesicle Cargo Release. *Nano Lett.* **11**, 888–892 (2011).

9. Kedenburg, S., Vieweg, M., Gissibl, T. & Giessen, H. Linear refractive index and absorption measurements of nonlinear optical liquids in the visible and near-infrared spectral region. *Opt. Mater. Express, OME* **2**, 1588–1611 (2012).

10. Smith, B. E., Roder, P. B., Zhou, X. & Pauzauskie, P. J. Hot Brownian thermometry and cavity-enhanced harmonic generation with nonlinear optical nanowires. *Chemical Physics Letters* **639**, 310–314 (2015).

11. Roder, P. B. *et al.* Photothermal Superheating of Water with Ion-Implanted Silicon Nanowires. *Advanced Optical Materials* **3**, 1362–1367 (2015).

12. Bird, R. B., Stewart, W. E., Lightfoot, E. N. & Klingenberg, D. J. *Introductory Transport Phenomena*. (Wiley, 2014).

13. Roder, P. B., Smith, B. E., Davis, E. J. & Pauzauskie, P. J. Photothermal Heating of Nanowires. *J. Phys. Chem. C* **118**, 1407–1416 (2014).

14. Mylona, S. K. *et al.* Reference Correlations for the Density and Viscosity of Squalane from 273 to 473 K at Pressures to 200 MPa. *Journal of Physical and Chemical Reference Data* **43**, 013104 (2014).

15. Son, J. S. *et al.* Large-Scale Synthesis and Characterization of the Size-Dependent Thermoelectric Properties of Uniformly Sized Bismuth Nanocrystals. *Angewandte Chemie International Edition* **50**, 1363–1366

16. Mingo, N., Yang, L., Li, D. & Majumdar, A. Predicting the Thermal Conductivity of Si and Ge Nanowires. *Nano Lett.* **3**, 1713–1716 (2003).

17. Draine, B. T. & Flatau, P. J. User Guide for the Discrete Dipole Approximation Code DDSCAT 7.3. *arXiv:1305.6497 [astro-ph, physics:cond-mat, physics:physics]* (2013).

18. Yan, C. & Lee, P. S. Bismuth-Catalyzed Growth of Germanium Nanowires in Vapor Phase. *J. Phys. Chem. C* **113**, 2208–2211 (2009).

19. Niklasson, G. A., Granqvist, C. G. & Hunderi, O. Effective medium models for the optical properties of inhomogeneous materials. *Appl. Opt., AO* **20**, 26–30 (1981).

20. Berg-Sørensen, K. & Flyvbjerg, H. Power spectrum analysis for optical tweezers. *Review of Scientific Instruments* **75**, 594–612 (2004).

21. Simpson, S. H. & Hanna, S. Stability analysis and thermal motion of optically trapped nanowires. *Nanotechnology* **23**, 205502 (2012).

22. Comuñas, M. J. P. *et al.* Reference Correlation of the Viscosity of Squalane from 273 to 373 K at 0.1 MPa. *Journal of Physical and Chemical Reference Data* **42**, 033101 (2013).

23. Hagemann, H.-J., Gudat, W. & Kunz, C. Optical constants from the far infrared to the x-ray region: Mg, Al, Cu, Ag, Au, Bi, C, and Al_2_O_3_. *J. Opt. Soc. Am., JOSA* **65**, 742–744 (1975).

24. Aspnes, D. E. & Studna, A. A. Dielectric functions and optical parameters of Si, Ge, GaP, GaAs, GaSb, InP, InAs, and InSb from 1.5 to 6.0 eV. *Phys. Rev. B* **27**, 985–1009 (1983).
