# Supplementary material for: Optically oriented attachment of nanoscale metal-semiconductor heterostructures in organic solvents via photonic nanosoldering
Source: Nat Commun. 2019 Oct 30;10:4942. doi: 10.1038/s41467-019-12827-w (PMC6821866; doi:10.1038/s41467-019-12827-w)
Supplement: Supplementary file 6 — Description of Additional Supplementary Files [file 41467_2019_12827_MOESM6_ESM.docx]

**Title:** Supplementary Movie 1
**Description:** Video demonstrating the optical trapping of colloidal bismuth-seeded germanium nanowires in an organic solvent dispersion.

**Title:** Supplementary Movie 2
**Description:** Video demonstrating the optically oriented assembly of an extended, periodic, bismuth-nanocrystal/germanium-nanowire heterostructure via the optical trapping and photonic nanosoldering of colloidal bismuth-seeded germanium nanowire building blocks.

**Title:** Supplementary Movie 3
**Description:** Video showing an optically trapped, superheated bismuth-seeded germanium nanowire being intentionally brought into contact with the glass coverslip of the trapping chamber.
